# Editorial on “Developing Benchmarks in the Diagnosis and Treatment of Pulmonary Arterial Hypertension in a Tertiary, Academic Medical Center”

**DOI:** 10.1002/pul2.70081

**Published:** 2025-04-15

**Authors:** Robert P. Frantz

**Affiliations:** ^1^ Department of Cardiovascular Medicine Mayo Clinic Rochester Minnesota USA

Kholdani and colleagues describe patient characteristics, completeness of diagnostic testing, treatment patterns, risk characteristics, and outcomes of a large cohort of patients with pulmonary arterial hypertension seen at an academic pulmonary hypertension center over a 20‐year period [1]. One of the goals of this project was to ascertain the extent to which guideline recommended diagnostic testing is actually being performed. There are several notable aspects of this analysis.

Right heart catheterization, the cornerstone of diagnosis in PAH, frequently lacked critical components when performed outside of the referral center. Measurement of cardiac output was deemed missing in 23% of cases.

For patients where cardiac output had been measured but PVR not calculated, often it could be calculated based upon the available data, but was not included in the catheterization report. This may reflect lack of automated programs in some cath lab systems that would perform this calculation. Although the calculation of PVR is not complex, this writer has seen a patient where the provider at the referring center had improperly calculated the PVR at greater than 6 Wood units, the patient was started on dual oral PAH therapy without benefit, and upon referral to our center it was recognized that the PVR was actually less than 2 Wood units. In our experience other errors, particularly incorrect pulmonary artery wedge pressure measurements, are quite common. In the current report, only 38% of patients for whom acute vasodilator testing is guideline recommended actually had it performed on RHC outside of the referral center. The combination of these findings reinforces current guideline recommendations for fast‐track referral of patients with suspected PAH and chronic thromboembolic pulmonary hypertension (CTEPH) to a referral center before RHC, and then to perform the RHC at the referral center [2]. Heeding this recommendation would expedite evaluation and treatment and reduce cost of care and patient burden of testing by avoiding frequent need to repeat the RHC at the referral center.

Ventilation/perfusion testing to rule out thromboembolic pulmonary hypertension remains underperformed, though this is improving. It is conceivable that this improvement reflects in part the emphasis placed on it by reviewers from the Pulmonary Hypertension Association (PHA) reaccreditation committee, and is encouraging to see, given the fundamental importance of differentiating chronic thromboembolic pulmonary hypertension from pulmonary arterial hypertension.

Frequency of assessment for sleep disordered breathing also appears to be suboptimal. The notably high prevalence of sleep disordered breathing in PAH, and its association with severity of RV dysfunction and survival [3] mandates that such assessment, starting with a simple tool such as overnight oximetry, be systematically performed.

The prevalence of PAH related to drugs and toxins is also quite notable, representing 35% of PAH patients in the most recent era of the Stanford cohort, and another reminder of the societal need to continue to address the epidemic of drug abuse including methamphetamine that is associated with development of PAH.

The very process of looking at frequency of such testing permits process improvement; what we do not know we cannot change. This is a great strength of the PHA‐Accredited PH Care Center program that accredits centers with expertise in pulmonary hypertension. There are nearly 100 accredited centers in the United States; the accreditation process carefully examines practice patterns and adequacy of testing at these centers, allowing each center to understand their current state and facilitate ongoing improvement. These centers also participate in the PHA Registry, and the numerous publications from this registry provide an excellent portrait of characteristics, therapy, and outcomes of patients with pulmonary hypertension across these centers. As an example, a recent PHAR publication [4] described overall 3‐year mortality of 21% in the PHAR registry, quite similar to the 17% 3‐year mortality in the Stanford cohort. The persisting relatively high morbidity and mortality of pulmonary arterial hypertension is sobering.

Through self‐examination comes the opportunity for improvement. By shedding light on practice patterns and outcomes of PAH patients at a major academic pulmonary hypertension center, Kholdani et al. have provided the pulmonary hypertension community with an admirable roadmap for ongoing quality improvement initiatives at other centers who aspire to excellence in the assessment and care of complex PAH patients.

## Author Contributions

Robert P. Frantz is sole contributor.

## Ethics Statement

The author has nothing to report.

## Consent

The author has nothing to report.

## Conflicts of Interest

The author declares no conflicts of interest.
